# Supplementary material for: Are carbon emissions trading and green financial instruments synergistic? -Comprehensive quantitative research based on content analysis
Source: PLoS One. 2024 Mar 7;19(3):e0298601. doi: 10.1371/journal.pone.0298601 (PMC10919601; doi:10.1371/journal.pone.0298601)
Supplement: S1 Data — (ZIP) [file pone.0298601.s001.zip › Data/地级市Z_F工作报告.zip!/║╙▒▒/└╚╖╗╩╨/└┤╫╘╣1⁄2╓┌║┼í╛┬φ┐╦╩2╛▌═°í┐_2018.docx]

2017年工作回顾

2017年，是我市发展历史进程中极不平凡的一年。这一年，大事多、喜事多，党的十九大胜利召开，京津冀协同发展深入实施，北京城市副中心、北京新机场加快建设，雄安新区横空出世，使我们迎来了千载难逢的历史性窗口期和战略性机遇期。这一年，难事多、干事多，我们坚决落实国家和省关于京冀交界地区历史上最严格的管控政策，强力推进“无钢市”“禁煤区”“北方地区冬季清洁取暖试点城市”建设，强势开展大气污染防治综合治理攻坚行动，全面打响蓝天保卫战，我们经历着转型升级、爬坡过坎的关键期和阵痛期。可以说，机遇与挑战同在，希望和困难并存。认真落实习近平总书记视察河北时的重要指示要求，在服从服务京津冀协同发展中加快发展自己成为全市经济社会发展的大格局和新常态。

一年来，在市委的坚强领导下，在市人大、市政协监督支持下，全市上下坚持以习近平新时代中国特色社会主义思想为指导，深入学习宣传贯彻党的十九大精神，认真落实执行省委、省政府重大决策部署，抢抓机遇、迎难而上、苦干实干、砥砺奋进，紧盯年初既定工作目标不动摇，狠抓各项任务落实不松劲，整体工作保持了平稳发展态势。2017年《政府工作报告》中确定的129项重点工作基本完成。主要经济指标平稳增长。地区生产总值完成2880.6亿元，同比增长6.8%。全部财政收入完成673.1亿元，同比增长13%；一般公共预算收入完成331.5亿元，可比增长9.8%。固定资产投资完成2631.8亿元，同比增长7%。社会消费品零售总额完成980.4亿元，同比增长11.2%。城乡居民人均可支配收入分别达到37474元和15487元，增长8.2%和8.4%。

(一)协同发展取得新进步。严格落实管控要求。坚决落实中央和省对京冀交界地区和雄安新区周边地区的管控政策，北三县严格执行与北京城市副中心“统一规划、统一政策、统一管控”要求，积极配合编制通州与北三县整合规划；环京六县(市、区)和环雄安新区周边县(市)实施最严格的规划、人口、产业、生态和房地产等管控措施，全市域除大城县外商品房实施严格限购。积极做好地方服务工作。配合编制北京新机场临空经济区总体规划，扎实做好北京新机场供油、空管工程等项目建设地方工作；全力完成白家务水源地迁建、天堂河改移工程；实质启动和扎实推进新机场回迁区安置房建设，依法明确建设主体，建立健全相关机制，完成了村庄拆迁，具备了全面开工建设的条件。主动做好京雄、廊涿、城际铁路联络线等轨道交通和新机场北线、唐廊等高速公路项目前期工作，津石高速廊坊段开工建设。主动加强对标对接。强化规划对接，聘请编制北京新机场临空经济区规划、雄安新区规划的知名专家和设计团队，对城市总体规划进行修编。强化产业对接，严格落实《河北省京冀交界地区新增产业的禁止和限制目录》，严禁承接和布局一般性制造业、中低端第三产业项目。强化高端项目对接，中安信碳纤维一期正式投产，中信国安影视中心建成投用，京东跨境电商保税区北方中央仓、中关村互联网文创廊坊园加快建设；大兴、北辰、廊坊“一十百千万”教育协同工程深入推进，永清北大附属廊坊益田实验学校开工建设，京廊中医药协同发展“8·10”工程深度合作。强化主城区功能对接，强势启动30个城中村、9个危陋住宅区拆迁改造，拆除面积1.6万亩，为优化交通、产业布局、大气污染防治、提高居民生活水平提供了新空间。实施了艺术大道贯通、外环路西南环线闭合及11条断头路打通工程，完成了金光道、和平路等11条道路提升改造。新增大热电集中供热面积480万平方米，大热电集中清洁供热面积达到总集中供热面积的42.1%，集中供热和清洁能源供暖率达到100%。生活垃圾直运二期工程和公厕垃圾中转站提升改造工程竣工，生活垃圾焚烧发电二期扩建项目和杨税务垃圾山治理工程扎实推进。静脉产业园、市殡仪馆完成选址。万庄、铁路北、凯发新泉二期3座污水处理厂建成通水调试，主城区日污水处理能力将达到21万吨，可满足未来五年污水处理需求。南水北调配套工程全部完成，主城区清泉地表水厂建成投用，主城区及沿线五县(市)受水区全部切换长江水，主城区日均有效减采地下水10万吨。主城区完成环卫体制改革重心下移，实现主次干道与小街小巷一体化管理。县城建设得到加强，大厂县获得国家级园林县城称号，文安县等7个县城被评为省级文明城。美丽乡村建设扎实推进，104个村庄被评为省级美丽乡村。着力推动区域协同。与通州区、武清区共同签署“通武廊”协同发展战略合作框架协议，共同组建了10个基础教育协同共同体，开发了3条旅游精品线路，实现了区域气象数据信息共享，首批“通武廊”人才服务绿卡正式颁发。北京亦庄·永清高新区等7个园区和载体项目被“京津冀三地协同办”列入京津冀产业转移承接重点平台。深化京津廊生态环境联防联控联治，共同推进大运河保护和生态建设，“平三蓟”京津冀国家生态文明先行示范区建设稳步推进。

(二)发展方式实现新转变。坚定不移去产能。做到讲政治停产能，在市场价格跳跃回升形势下，落实上级要求不动摇不含糊，通过“政府推动、企业主体、市场运作、政策支持”的市场化模式，整体退出2家钢铁企业，消化产能1000万吨，得到省委、省政府的充分肯定。做到强环保去产能，严格落实国家和省治理工业企业污染要求，全市“散乱污”企业治理12003家，关停取缔数量达到86.2%，六大高耗能行业增加值下降27.3%。做到求效益去产能，加大传统优势产业整合提升力度，文安县近3000家胶合板生产企业整合保留200多家，5家企业参与国家行业标准制定；大城县绝热节能材料产业竞争力大幅提升，年产量占到全国市场份额的60%以上；霸州市下游近800家涉钢企业加快转型，产业链末端2700余家“散乱污”企业完成治理。扎实推进农业种植结构调整，调减籽粒玉米、棉花、油料种植面积29.5万亩，新建高端设施蔬菜32万亩；新增市级以上现代农业园区13家、省级农业产业化龙头企业9家，农业产业化经营率增速继续保持全省首位。聚焦重点增动能。进一步加大技术改造力度，全年工业技改投资完成772.4亿元，占工业投资比重达到60.8%；实施技改项目590项，列入省千项计划76项，固安县成功争列首批省工业转型升级试点示范县。战略性新兴产业支撑力进一步增强，增加值占规上工业增加值比重达到16.2%，创历史新高；华为云计算中心三期、京津冀大数据应用展示体验中心建成投产，第六代AMOLED项目开始安装设备，中国中冶总部基地正式运营，香河通航产业园已完成民航局场址批复。优化项目投资结构，在实施的33项省重点项目和100项市重点项目中，战略性新兴产业项目分别达到51.5%和36%，11项新开工省重点项目，完成投资265亿元，投资到位率338%，拉动全市固定资产投资增速提高7.6个百分点。推进创新添势能。着力提升科技创新能力、企业创新能力、协同创新能力，新增科技型中小企业1437家、科技小巨人企业77家、高新技术企业126家；新增省级院士工作站10家，居全省第1位，新认定市级孵化器和众创空间21家；全市技术合同交易额达24亿元，同比增长13.8%，专利申请量达到6292件，科技进步贡献率达到55%。三次产业结构由7.3∶44.2∶48.5调整到6.5∶43.8∶49.7，三产占比居全省第2位。现代服务业实现增加值920亿元，占服务业比重达到64.2%，对经济增长的贡献率超过80%；旅游业总收入达到372.7亿元，同比增长33.2%。“大智移云”产业首位度持续提升，电子信息产业营业收入及增速、实现利润、上缴税金均居全省首位。

(三)空气质量得到新提升。坚持科学治霾，持续深化与PM2.5专家组合作，成立由院士牵头的智慧环境生态研究院，建立完善空气质量监控大数据平台。推进环保工作重心下移，147个乡镇(街道)、园区全部设立环保所，90个乡镇全部设立空气质量监测站，在全省率先实现乡镇监测全覆盖。在市主城区建立联防联控协调联动机制，有效解决了三区和市直部门各自为战、合力不强的问题。完成环保垂直管理制度改革。持续开展“利剑斩污”执法行动，检查各类企业3.4万家次，行政处罚金额1.25亿元，有效打击了环境违法行为。强化精准治标，建立严格的文明施工监管机制，对主城区135个建筑工地全部安装视频监控和在线监测系统，全面落实建筑工地扬尘防治“七个100%”。针对不同季节及主次路段实时状况，科学制定路面湿扫、洒水及雾炮作业方案，扬尘治理达标率达到100%。对拆迁垃圾实行限时、限路、限车，交警、综合执法局全程跟踪监管的清运模式。大力整治露天烧烤，基本实现市区无露天烧烤摊点，无店外摆桌经营。加强祭祀品焚烧管控、秸秆禁烧，运用市场化手段推进秸秆综合利用。市政府出台《廊坊市烟花爆竹安全管理办法》，强化禁放管控的法制保障。推进深入治本，能源结构、产业结构、交通结构不断优化。在农村完成61.4万户“气代煤”、8.1万户“电代煤”工程，占到全省近三分之一，基本实现农村地区散煤清零目标。提标改造35蒸吨以上燃煤锅炉76台，淘汰改造35蒸吨以下燃煤供热锅炉194台，彻底取缔10蒸吨以下燃煤锅炉4175台。全面完成“散乱污”企业整治，并建立起防反弹常态机制，环保部京津冀及周边地区“散乱污”整治暨秋冬季攻坚阶段总结现场会在我市召开。省定158家涉VOCs排放企业得到全面治理。主城区购置新能源公交车100辆，对运营卡车实施证卡管理、错峰通行、卡点分流。恢复和改造提升湿地1.45万亩，植树造林26.1万亩，森林覆盖率达到30.66%。2017年，全市空气质量在全国74个国控城市中倒排第13位，稳定退出“倒排前十”的基础进一步夯实。PM2.5年浓度为60μg/m3，超额完成年度62μg/m3的目标。在全省142个县(市、区)空气质量排名中，我市9个县(市、区)进入前20名。特别是第四季度，全市PM2.5平均浓度为54μg/m3，同比下降45.45%，浓度和削减率在“2+26”城市中分别正排第2和第3位，优良天数增加了21天，空气质量达到近年同期最好水平。人民群众明显感觉到天更蓝了、空气更清新了，蓝天幸福感越来越强了。

(四)营商环境得到新优化。进一步加大改革力度，实施国家级试点19项、省级试点30项，充分发挥试点在改革全局中的示范带动作用。国家中小城市综合改革试点任务顺利完成，形成园区共建模式创新、土地弹性出让制度改革等16项改革成果；石保廊全面创新改革试验区扎实推进，开发区改革、收入分配制度改革等7项重点工作取得阶段性成果。规范组建市县两级行政审批局，市级划转承接审批事项169项，审批人员由240多人减至70人，压减审批环节77项，推出当日办结事项54项、企业登记便利化26条等改革措施，平均提高审批效率68%；衔接落实省取消下放行政审批事项32项，全面实行“三十八证合一”，在全省率先实施跨部门“双随机、一公开”联合抽查，积极推行“互联网+政务”在线审批模式，群众和企业满意度有了新提升。做优做大开放平台，“5·18”经洽会在场馆建设、会务组织、布展方式等方面按照省委、省政府要求实现了“革命性变化”；“9·26”农交会市场化、品牌化、专业化水平实现新突破；首次承办全国第二十七届图书交易博览会，展位数量、观展人数、销售金额创历届之最。支持企业“走出去”，华夏幸福、宏泰发展等企业在印尼、德国等国家建设产业新城和创新中心。廊坊综合保税区获得国务院正式批复，将成为我市开放度和自由度最高的开放平台。不断优化服务环境，积极开展企业帮扶，制定《关于强化重点企业联系服务机制实施方案》，建立领导分包服务、银企对接等联系服务机制，及时解决企业反映的163个问题；开展营商环境集中整治行动，实施降低制度性交易成本、规范政务运行等八项重点攻坚，企业反映普遍、社会影响较大的222个热点难点问题全部办结。全市新增市场主体6.4万户，市场主体总量达到32万户，增长19%。

(五)群众获得感有了新增强。全年财政支出用于民生方面的资金达到470.6亿元，同比增长11.9%，占财政支出比例达到80%。持续提升民生保障水平。从最困难、最弱势的群众做起，全面提高城乡低保、特困人员基本生活补助、困难残疾人生活补助、重度残疾人护理补贴、孤儿救助、低保家庭高中生补助6项社会救助标准，分别达到全省最高标准，其中3项与北京持平。城乡居民养老保险基本养老金平均达到123元，为全省最高。支出就业补助资金1.54亿元，新增城镇就业岗位6.6万个。在市级层面对2304个支出性贫困户、6113名贫困人口全部建档立卡，并出台精准脱贫攻坚行动实施方案，开展健康、助残、教育、危房改造、社保兜底、产业和就业脱贫扶贫攻坚行动；对口帮扶张家口5个深度贫困县任务取得阶段性成效。加快补齐教育卫生文化短板。全市启动建设中小学和幼儿园62所，完成23所、新增学位1.8万个，改善义务教育学校252所，建设标准化学校140个，补充教师2256名。顺利实施12家城市公立医院改革，取消药品加成，药价平均降低12.7%。建成基层综合文化服务中心2000个，城市社区和农村健身设施覆盖率分别达到95%和80%以上。市体育局被评为全国群众体育先进单位。全力保障社会安全稳定。进一步夯实安全监管基础，在市安委会20个成员单位分别设立安监科室；在全市147个乡镇(街道)、园区分别设立安监站；在市、县城市建设系统建立专门机构，专人专职负责“气代煤”质量监管；进一步明确了乡级安全监管机构，在农村规范组建了安全监管员和安全协管员队伍。深入开展安全生产大检查专项治理行动，工矿商贸领域事故发生起数、死亡人数均比2016年降低42.9%，安全生产形势保持总体稳定；市安监局被评为全国安全生产监管监察先进单位。集中解决历史遗留问题，“问题楼盘”化解率达到90.63%，全市违法占用耕地整改率达到98%，“一区三边”违法建设整治完成98%。为有效解决农民工欠薪问题、保障“煤替代”工程质量安全、冬季取暖用气保障、军队退役人员政策落实，坚持问题导向，分别成立工作专班，实行独立办公，及时解决各类问题，收到明显阶段性成效。强力推进“京畿平安示范区”建设，持续开展打击非法集资、传销、互联网金融诈骗等专项活动，圆满完成党的十九大、暑期安保等重大安保任务。我市获得全国社会治安综合治理“长安杯”、全国社会治安综合治理优秀市、全国未成年人思想道德建设工作先进城市、河北省双拥模范城、河北省文明城市等荣誉称号。民族宗教、外事侨务、人民防空、史志档案、气象地震、地理信息、援藏援疆、妇女儿童等各项工作均取得了新成就。

(六)政府自身建设实现新提高。牢固树立“四个意识”、坚定“四个自信”，认真学习贯彻党的十九大精神，坚决维护以习近平同志为核心的党中央权威和集中统一领导，扎实推进“两学一做”学习教育常态化制度化，自觉接受市委领导。持之以恒正风肃纪，认真落实党风廉政建设主体责任，严格执行中央八项规定及实施细则精神，在政府系统扎实开展“一问责八清理”、基层“微腐败”两个专项整治行动，查实问题16577件，追究直接责任人16852名。忠实履行宪法和法律赋予的职责，持续完善依法科学民主决策机制，行政执法三项制度改革试点取得实质性成果。自觉接受人大法律监督、工作监督和政协民主监督，重大事项向人大报告、向政协通报，办理答复人大代表建议291件、政协委员提案694件，办复率均为100%。市政府系统获得省级以上表彰65项。

2018年工作总体要求和主要目标

今年是贯彻党的十九大精神的开局之年，是改革开放40周年，是决胜全面建成小康社会、实施“十三五”规划承上启下的关键一年，做好今年工作意义重大。

今年政府工作的总体要求是：全面贯彻党的十九大精神，坚持以习近平新时代中国特色社会主义思想为指导，深入落实中央经济工作会议、省委九届六次全会和省十三届人代会精神，按照市委六届四次全会战略部署，坚持稳中求进工作总基调，坚持新发展理念，紧扣我国社会主要矛盾变化，按照高质量发展的根本要求，统筹推进“五位一体”总体布局和协调推进“四个全面”战略布局，以供给侧结构性改革为主线，推动质量变革、效率变革、动力变革，更加突出协同发展，更加突出转型升级，更加突出改革创新，更加突出生态支撑，更加突出民生改善，更加突出优化营商环境，更加突出防范化解重大风险和维护安全稳定，紧紧围绕与京津一体化发展的创新驱动经济强市定位，促进经济社会持续健康发展，为新时代加快建设经济强市、美丽廊坊而努力奋斗。

今年全市经济社会发展的主要预期目标是：地区生产总值同比增长7%左右，一般公共预算收入增长7%左右，固定资产投资增长8%左右，规上工业增加值增长3%以上，社会消费品零售总额增长11%以上，实际利用外资增长5%以上，城乡居民人均可支配收入分别增长7.5%和8%以上，居民消费价格指数涨幅控制在3%左右。

做好今年和今后一个时期的政府工作，必须旗帜鲜明讲政治。树牢“四个意识”，坚定“四个自信”，坚决维护和捍卫习近平总书记在党中央和全党的核心地位，坚决在思想上政治上行动上同以习近平同志为核心的党中央保持高度一致，始终坚持以习近平新时代中国特色社会主义思想武装头脑、指导实践、推动工作。必须坚定不移顾大局。深入推进京津冀协同发展战略，自觉服从服务北京城市副中心、北京新机场及临空经济区、雄安新区建设等国家重大决策，严格落实各项管控措施，紧紧扭住疏解北京非首都功能的“牛鼻子”，认真落实全省“抓好三件大事、打好六场硬仗、实施八项战略、深化九项改革”的工作要求，在服从服务中加快发展自己。必须全心全意为人民。坚持以人民为中心的立场。急群众之所急、想群众之所想、做群众之所需，把群众满意不满意、高兴不高兴、答应不答应作为一切工作的出发点和落脚点。必须毫不动摇补短板。坚持质量第一、效益优先，把高质量发展贯穿于经济社会发展各领域和全过程，让创新成为高质量发展的第一动力,让协调成为高质量发展的内生特点，让绿色成为高质量发展的普遍形态，让开放成为高质量发展的必由之路，让共享成为高质量发展的根本目的。紧扣社会主要矛盾的变化，坚持问题导向，着力解决发展速度与发展质量、经济发展与社会发展、城市与乡村、区域与区域之间等发展不平衡问题和创新动能、公共服务供给等发展不充分等问题，扭住幼、学、劳、病、老、住、弱“七个有所”重点，不断满足人民日益增长的美好生活需要。必须心无旁骛打基础。基础不牢地动山摇。前人栽树后人乘凉。树立正确政绩观，尊重经济社会发展规律，不驰于空想、不骛于虚声，保持战略定力和历史耐心，有守有为、有舍有得，久久为功、苦干实干，以钉钉子的精神、锲而不舍的执着，多做打基础、蓄后劲、利长远的利民实事，创造经得起实践、人民和历史检验的发展业绩。

2018年主要工作任务

围绕2018年工作总体要求和目标任务，重点做好六项工作：

(一)在更加注重协调上再聚焦，在打造京津冀交界区域高质量协同发展“桥头堡”上再发力

主动对接上位规划。按照建设世界级城市群重要节点城市要求，积极衔接京津城市总体规划和通州与北三县地区整合规划、新机场临空经济区总体规划、雄安新区总体规划，认真做好规划期至2035年的城市总体规划修编前期工作，尽快编制各类专项规划和总体城市设计，进一步调整优化城市功能和空间布局，实现与国家重大战略工程的协调发展、融合发展、错位发展。继续落实好中央和省对京冀交界地区、雄安新区周边地区的管控要求，持续对规划、土地、房地产等实施严管严控。对违法建设“零容忍”，坚决遏制增量，有效治理存量。建立健全惩防长效机制，对重点区域开展全面监测、专项清理。

持续深化与京津重点领域对接合作。加快推进交通互联互通，全面保障京雄、京唐、廊涿及城际铁路联络线廊坊段建设，大力推进天津至新机场联络线、平谷线前期工作，着力构建连通京津雄的快捷高效轨道交通网络；配合做好京秦、津石高速公路项目建设和京沪高速北延、唐廊、京德高速公路前期工作，北京新机场北线高速年内开工。努力提升生态环境协同治理水平，大力抓好永定河、潮白河、北运河、大清河等域内河流综合治理生态工程，严格落实上级关于区域生态信息共享和补偿机制，全面保护域内森林湿地绿地资源。积极承接北京非首都功能疏解，紧紧扭住非首都功能疏解“牛鼻子”，下大决心务实推进与北京在教育、医疗、文化等领域合作，加快实施与首都儿研所、北京佑安医院、人民大学、北京师范大学等合作项目，推动梦廊坊“丝绸之路”国际文化艺术交流中心今年投入使用；依托北京亦庄·永清高新区等7个产业转移承接重点平台，着力引进一批京津转移高端产业项目，力促中国中冶总部基地、中交建新能源汽车产业基地、京东集团广阳、永清电商物流等项目尽快开工。深入推进“通武廊”合作，携手打造协同发展试验示范区。

着力优化区域产业布局。坚持协同融合，统筹调整全市域产业布局，实现协调联动发展。支持北三县大力发展以智能制造、文化创意、总部经济等为重点的高端高新产业和现代服务业，支持主城三区和永清、固安超前谋划储备一批高端临空产业和新一代电子信息、新材料、航空航天等高新技术产业项目，支持南三县瞄准北京高端产业转移和产业链延伸，重点发展高端装备制造、新材料、节能环保等新兴产业，努力构建与京津和雄安新区分工明确、相互协调、融合发展的大产业格局。

持续提升协同发展承载能力。全面融入京津冀世界城市群建设，以打造智慧城市为目标，加快完善城市功能，推进行政区划调整，做大做强中心城市，提升城市空间承载力。今年是实施棚户区改造三年行动计划的第二年，在2017年棚户区改造工作基础上，再启动7个城中村和4个危陋小区改造项目。“5·18”前广阳道东延等12个“断头路”区段续建工程全部实现主路通车，新开工新开路北延、永兴路南延等5个区段；外环路西南环线、艺术大道贯通西延工程力争6月底前实现通车。高标准对廊涿引线、和平路、银河北路、广阳道、爱民道、金光道等6条道路和北吉里等119条小街巷进行提升改造，把廊涿引线打造成为精品迎宾线。加强与北京铁路局沟通协调力度，启动光明道上跨京沪铁路立交桥工程。加快停车场专项规划编制和建设，多渠道拓展1万个停车泊位，规范机动车便道停放秩序，着力解决“停车难”“乱停车”问题。提升市区公交服务能力，优化线路站点，力争再投放新能源公交车100部，争创全省优先发展公交示范城市；加快推进与京津实现公交“一卡通”。不断提高绿化亮化水平，加快外环路绿化景观带建设，实施艺术大道和西南环线高标准绿化工程，瑞丰公园力争“五一”前具备开园条件；继续实施广阳道等4条道路的夜景亮化，打造精品夜景示范街。谋划实施主城区“雨污分流”工程，启动永兴路南延配套3座泵站和龙河高新区2座泵站建设，解决污水处理能力不足的短板；杨税务垃圾山“五一”前务必根除；生活垃圾焚烧发电项目二期工程3月底建成投用；完成静脉产业园生活垃圾和餐厨垃圾处理项目主体工程。实施“厕所革命”，按照1平方公里3—4处的标准，实现主城区公厕全覆盖。加快广阳水库前期工作，年内开工建设，改变廊坊在全省没有地表水库的历史，为主城区用水提供最可靠的保障。全力推动北京新机场回迁安置区建设，力争一期工程年底竣工封顶。加快建设市民服务中心，力争年底前具备入驻条件。市殡仪馆年内主体竣工。进一步提升县城建设水平。加快推进安次北田曼城国际小镇等10个首批省级特色小镇建设，三河、香河、永清力争通过国家园林县城验收，大城确保通过省级园林县城验收。

(二)在更加崇尚创新上再聚焦，在争当京津冀交界区域高质量现代经济体系“生力军”上再发力

加快壮大新兴产业。壮大主导产业增实力。大力提升“大智移云”产业首位度，重点打造新型显示、智能制造、大数据、电子商务、文化创意等八大产业集群；依托京津冀大数据综合试验区建设，推进大数据产业与实体经济融合，支持润泽科技、华为、科大讯飞等龙头企业在我市加快发展，支持工惠驿家等大数据开发应用项目加快实施，支持“中鼎云”数据基地和京东华北云数据中心等项目加紧开工建设，力促第六代AMOLED、京东跨境电商保税区北方中央仓项目上半年投产，加快推进京津冀新能源汽车产业基地、郎创航天机械、央视影视传媒制作工场、深圳彩虹气雾剂园区等重点项目建设。支持河北(燕郊)军民融合科技孵化中心建设。狠抓有效投资育产业。深入实施项目建设“311”工程，申列省重点项目30个以上，安排市重点项目100个以上，建设亿元以上项目1000个左右，以高质量项目建设带动产业链集聚延伸、促进产业向高端发展，不断增强产业发展后劲。培育品牌提质效。以建设“全国质量强市示范城市”“知名品牌创建示范区”为契机，引导企业增品种、提品质、创品牌，参与国家和行业标准制定，争创国家和省级质量奖，提升“廊坊品牌”的竞争优势。持续加强政务诚信建设，健全守信联合激励和失信联合惩戒机制，引导企业以信誉赢得市场、赢得效益。

加快改造传统产业。抓技术改造。以智能制造为重点，培育“两化融合”“互联网+”制造业试点示范企业10家以上，实施技改项目500项，技改投资占工业投资比重保持在60%以上。抓工业设计。启动建设市工业设计中心，培育扶植一批专业化工业设计公司和机构，在家具制造、装备制造等行业开展制造环节整体设计和工业产品再设计，高水平办好我市首届工业设计周。抓产业整合。总结推广板材加工、保温建材等传统产业整合提升成功经验，支持家具制造、印刷包装等传统优势产业，通过异地搬迁、引入战略合作伙伴兼并重组、链式发展，向集团化、股份合作化、管理现代化跃升。抓产能退出。支持霸州加快推动钢铁企业退出后的产业转型、区域转型，尽快启动实质性工作。安次区、文安县要加快推进域内钢铁企业的退出工作。加大低效用地再开发力度，严格执行质量、环保、能耗、安全等法规标准，运用市场化、法治化手段，对不符合规划、环保和产业政策的企业实施“腾笼换鸟”。

着力加快创新驱动发展。围绕建设国家创新型城市，深入实施创新驱动发展战略，进一步完善“1+4+N”创新政策，强化各类要素支撑，持续激发“双创”活力。加快石保廊全面创新改革试验区建设，出台支持引进优秀人才的突破性政策，实施“十百千”工程，力争引进十名院士级人才、百名硕博士、千名本科大学毕业生来廊创新创业。设立“大智移云”产业发展基金，对“大智移云”领域的创新人才、创新项目，给予重点支持。推进京南科技成果转移转化示范区等国家级平台建设，廊坊开发区、北京亦庄·永清高新区、京南·固安高新区、霸州经济开发区分别引进和建设10家以上高端孵化器、众创空间和创客新城，实质性启动廊坊·中关村软件园人才与产业创新基地等协同创新基地，加速推进“京津研发、廊坊孵化转化产业化”。全年新增高新技术企业80家、科技型中小企业1000家以上；市级以上重点实验室、产业技术研究院、企业研发机构总量增长10%；各类孵化场地面积超过160万平方米；引进和转化京津科技成果产业化项目50个，全市技术合同交易额达到20亿元；专利申请量增长10%，万人发明专利拥有量达到4.8件。

着力实施乡村振兴战略。加快推动农业全面升级。制定实施农业供给侧结构性改革三年行动计划，深入开展“农业质量提升年”活动，加快发展高端设施、高效生态、休闲旅游等都市现代农业，推进农业由增产导向转向提质导向。由市农业局牵头组织制定标准化生产、农产品质量安全追溯体系，抓好农产品质量安全示范县建设，着力打造农产品品牌，农产品抽检合格率保持在98%以上，新增绿色有机农产品认证企业3家。加快推动农村全面进步。持续推进美丽乡村建设，实施农村人居环境整治三年行动计划，开展垃圾治理等专项行动。推进农村“厕所革命”，实现农村无害化卫生厕所全覆盖；加快“四好农村路”建设，制定三年实施计划，全年新改建农村公路300公里以上，探索建立县、乡、村三级路长制，市县乡管理机构设置率、人员编制到位率、农村公路列养率均达到100%，城乡客运公交化运行率达到60%以上。确保完成农村承包地确权登记颁证工作。加快推动农民全面发展。实施产业兴村强县行动，培育壮大特色产业集群，促进农民就近就地转移就业增收；开展城乡一体化公共就业服务，深化与京津劳务协作，增加农民工资性收入；引导承包地经营权有序流转，发展多种形式股份合作，增加农民财产性收入；落实和完善农业支持保护补贴等政策，增加农民转移性收入。抓好新型职业农民培育，加强农村思想道德建设，培育文明乡风、良好家风、淳朴民风。

(三)在更加倡导绿色上再聚焦，在构筑京津冀交界区域高质量生态建设“新高地”上再发力

坚决打赢蓝天保卫战。强化顶层设计，制定实施新一轮大气污染防治作战计划，依托大数据污染源解析，深化靶向治理、精准攻关。强化重点攻坚，统筹抓好秋冬季大气污染综合治理，深入落实省“1+18”政策体系，全面完成控煤、控车、控尘、控工业污染等目标任务。3月底前完成“散乱污”企业再核查整治，坚决杜绝死灰复燃、反弹回流；5月底前完成主城区350家涉VOCs企业的深度治理、分类施治、长效管控。加强与华北油田合作，加快实质启动杨税务潜山气田勘探开发建设。8月底前实现全域54台35蒸吨及以下燃煤锅炉清零。加强重型卡车管控，加快老旧车辆淘汰，科学规划道路禁限行交通疏导，继续实施柴油重卡禁限行措施。持续强化扬尘治理、秸秆禁烧等日常管控。落实省政府要求，将环保部门列为行政执法机构，在经费、编制、装备等方面强化保障，切实提升环保执法能力。

强力推进水污染治理。严格落实国家“水十条”和省“水五十条”，深入推动“河长制”，编制落实一河一策方案，以设立省级河长的5条河道、设立市级河长的8条河道为重点，实施河渠清理专项行动。参照大气治理模式，建立市水办统筹协调、各职能部门分口指导、各县(市、区)属地负责的联防联治机制。建立严格的河流断面水质生态补偿机制，量化水质监测、考核、补偿标准，对不达标河流下游地区实施生态补偿。加强入河排污口监管和河流断面监测，按照重点河流水质达标方案，持续推进重点河流河道治理和生态修复工程，确保泃河、潮白河、北运河、龙河、大清河、子牙河达到考核要求。确保13座在建污水处理厂全部按时完成、如期投用。加大环城水系治理力度，完成六干渠、八干渠黑臭水体治理任务，市区水系6月底前全面还清，彻底还群众一方碧水。

持续强化生态修复。革命性推进国家森林城市创建，以北京城市副中心、北京新机场、雄安新区边界地区生态修复为重点，启动大规模、高档次、新机制国土绿化攻坚行动，实施“一十百千万”工程。全年营造林100万亩，其中新造30万亩，全市森林覆盖率达到31%以上。深入落实国家“土十条”和省“土五十条”，以保护农用地和建设用地土壤环境为重点，提升土壤环境承载力，促进土壤与大气、水污染协同治理。积极打造北部运河文化水生态板块、中部永定河水生态文化板块、南部洼淀文化水生态板块。启动生态保护红线区域勘界定标，加强湿地修复与保护。

(四)在更加厚植开放上再聚焦，在建设京津冀交界区域高质量改革开放“示范区”上再发力

扎实推进重点领域改革。深化行政审批制度改革，及时推进取消和下放行政权力事项，扎实做好行政许可中介服务事项清理规范，进一步推动“互联网+政务服务”平台建设，将网上政务服务向基层延伸。深化商事制度改革，加大多部门联合“双随机、一公开”抽查力度和频次，推进“多证合一”扩容升级；开展“减证便民”专项行动。深化财税金融体制改革，加快壮大政府投资基金规模，充分发挥财政资金引导作用。切实做好环保税征收工作。完善企业上市融资扶持政策，引进、发展一批金融机构，提升中小企业贷款网上对接平台效能，推进农信社改制农商行，建立金融机构差异化考核评价体系，增强金融服务实体经济能力和效果。深化投融资体制改革，全面实施市场准入负面清单制度，促进社会资本和外资扩大投入。深化国资国企改革，稳步推进市属经营性国有资产集中统一监管，加快国有企业公司改革。积极推进农业农村、科教文卫等领域改革。切实打好优化营商环境硬仗，认真落实省《优化营商环境条例》，营造依法保护市场主体合法权益的法治环境、公平竞争的市场环境、鼓励创业的社会环境。企业家是财富的创造者、创新活动的实践者，要尊重、支持和爱护企业家，激发和保护企业家精神，构建亲清新型政商关系。大力支持本土企业家热爱家乡、发展家乡，真诚欢迎外埠企业家投资廊坊、共谋发展，使广大企业家感受到亲商、安商、富商的廊坊温度。

加快开发区改革发展。继续推动开发区体制机制创新，持续深化“四项改革”，支持符合条件的省级开发区设立“一级财政”，激发创新发展的动力和活力。要精准确定产业定位，实施精准招商，聚焦1—2个主导产业，着力建链补链强链，打造一批高端企业聚集、产业链条延展的合作产业园，实现全市开发区高质量发展。廊坊开发区要高标准建设好廊坊综合保税区，用足用好国家政策，积极引进高端产业和贸易项目，大力发展保税加工、保税物流、保税服务，打造我市经济发展新的增长点。

全面扩大双向开放。高水平办好今年“5·18”经洽会、“9·26”农交会等大型展会，加强与世界500强公司、大型央企和知名民企合作，下大力引进高端项目、区域总部。积极争创国家跨境电子商务综合试验区，依托京东跨境电商项目，打造我市进出口贸易的新业态、新亮点。深度融入“一带一路”，支持优势企业“走出去”，开展国际产能合作，并购国际资源和先进技术、研发团队，建设境外产业园区和技术创新中心。

(五)在更加推进共享上再聚焦，在勇当京津冀交界区域高质量全面建成小康社会“领头雁”上再发力

扎实推进精准扶贫脱贫。实施健康脱贫行动。尽快建立县域内住院先诊疗后付费和一站式报销服务制度，提供“六个一”免费健康服务，通过大病救助基金、商业保险等模式，切实解决大病患者医保报销范围外自付费用较高的问题。实施危房改造脱贫行动。对663户住房困难家庭，逐户提出改造、新建计划，确保到2019年全部完成建档立卡贫困户危房改造任务。实施产业帮扶和就业脱贫行动。对有创业就业需求的家庭，全面落实职业技能培训、创业培训，引导进入合作社、园区、龙头就业，年底前实现产业就业扶贫全覆盖。实施教育脱贫行动。在落实三免一助等省级政策的基础上，加快出台福彩助学等市级政策，确保不发生因贫辍学问题。实施助残脱贫行动。在严格执行残疾人两项补贴政策基础上，对生活特殊困难的低收入残疾人给予应急救助。实施社保兜底行动。对完全或部分丧失劳动能力的贫困人口，按规定纳入低保和特困救助范围，鼓励有条件的县(市、区)进一步提高低保标准，做到应保尽保。坚持“既不降低标准，也不吊高胃口”，严格建档标准和工作程序，完善动态调整及退出机制，做到应纳尽纳、应退尽退。

加强社会保障体系建设。积极落实省下达我市的各项社保扩面任务，继续提高企业和机关事业单位人员基本养老金，严格落实城乡低保等社会救助动态管理机制，切实做到应保必保、防止骗保。全力保障高校毕业生、农村转移劳动力、去产能职工、就业困难人员等重点群体就业，新增城镇就业岗位5.35万个，高校毕业生就业率保持在90%以上，城镇登记失业率控制在4%以内。坚持“房子是用来住的，不是用来炒的”定位，建立多主体供给、多渠道保障、租购并举的住房制度，通过大力发展租赁市场、棚改安置、新建商品房配建公租房和人才公寓等方式，满足群众多层次住房需求。

提升社区治理水平。严格落实城市小区非营利性配建中小学、幼儿园、停车场、卫生站、居委会办公场所、社区养老服务用房政策，对部门联审联验机制实行公开公示，切实解决应建未建、应交未交等问题。出台物业管理办法，建立健全物业服务企业市场化竞争机制，形成街道办事处、社区居委会、业主委员会、物业公司、社区警务室“五位一体”的物业管理模式。特别要下大决心彻底解决31个无物业老旧小区安全无保障、垃圾无人清、设施无人修、停车无秩序等问题。

坚持优先发展教育。促进教育均衡，严格落实县(市、区)学校建设主体责任，合理规划和建设义务教育学校，优先解决教育用地和资金问题，开通绿色通道，严格执行新建住宅区配建中小学幼儿园规定。在实施好去年续建项目的基础上，新改扩建中小学、幼儿园60所，其中主城区续建14所、改扩建4所、新建13所。加快推进廊坊一中迁建工程，确保秋季投入使用；续建改建管中一小、二小。广阳区新建续建中小学6所、幼儿园4所。安次区新建续建中小学10所、幼儿园4所。廊坊开发区续建中小学3所、幼儿园1所。在全省率先完成全面改薄工程，标准化学校创建率达到100%，学前三年毛入园率达到90%以上。提高教育质量，开展督政督学和评估监测，督导督查县(市、区)履职尽责。加强新时代教师队伍建设，推进教师编制市级统筹，通过增加编制、合理调配、购买服务等方式，补充教师2535名，强化绩效管理，不断提高教育教学质量。支持廊坊一中打造省内一流高中。深化产教融合，全面提升应用型、技能型人才培养，年内输送1万人，形成与我市现代产业体系相匹配、与充分就业相适应的现代高等教育、职业教育体系，为战略性新兴产业和现代服务业提供人才支撑。保障校园安全，依法彻底取缔无证幼儿园，严厉整治无证校车，全面推进校园安全网格化管理，持续开展学校、幼儿园周边安全整治行动，最大限度消除各类校园安全隐患，确保每个孩子安全上学、安心读书。

推进“健康廊坊”建设。以建设“健康家庭”为核心，构建覆盖全业务、全人群、全生命周期的健康照护模式。健全医疗服务体系，加快推进市人民医院、中医院、三院、中心血站及管道局医院改扩建工程，鼓励社会资本兴办医疗和健康服务机构，在全省率先建设12个“医体结合”国民体质监测与健康促进服务中心。推进医药卫生体制改革，大力推进主城区紧密型医联体、县城医共体、跨区域专科联盟建设，扎实推进家庭医生签约服务，依托“新时期赤脚医生”培训项目，加强基层签约医生团队建设，签约覆盖率达35%以上。继续做好计划生育工作，改革完善计划生育服务与管理。加快建设智慧卫生体系，推进全国第三批“电子健康卡”试点建设，争取国家健康医疗大数据华北中心落户廊坊。

大力发展文化旅游体育事业。深入实施文化强市工程，加快推进市非遗馆、美术馆、科技馆以及1000个基层综合性文化服务中心实质性建设，积极创建国家文化消费试点城市和全国版权示范城市，全力办好中国图书馆年会，启动“阅读在身边”工程，塑造书香城市品牌。推进大运河文化旅游带建设，加快全域旅游发展，开好全市旅游产业发展大会。全面推进体育惠民行动，启动社区健身设施提标工程，农村健身设施力争实现全覆盖，着力构建15分钟健身圈；启动市滑冰馆建设；举办好高水平足球赛事。

(六)在更加讲政治上再聚焦，在全力当好首都政治护城河“排头兵”上再发力

坚决守住安全生产底线。严格落实安全生产责任制，建立市、县两级安全生产监管责任清单，坚决落实安全生产一票否决制度。建立健全人防、技防、物防安全监管体系，县(市、区)安委会成员单位设立安监科，全面落实147个乡镇(街道)、园区安监站“三定方案”；建立健全安全监管信息化指挥调度平台和实时监测平台；在经费、车辆、设备等方面持续强化保障。实施“煤替代”工程安全监管队伍职业化、专业化管理，建立智能化安全综合监管平台，切实保障群众安全温暖过冬。深入开展安全生产隐患大排查大整治大问责“百日攻坚”行动，加强工矿商贸、道路交通、特种设备、消防等重点领域安全隐患专项排查整治，掀起全市“安监”风暴，坚决遏制重特大安全事故。着力提升食药安全监管水平。全面创建省级食品安全示范城市，强化基础建设和市场监管，探索建立重点产品电子追溯系统，全力保障人民群众“舌尖上的安全”。

强力推进平安廊坊建设。深入开展扫黑除恶专项斗争，聚焦涉黑涉恶问题突出的重点地区、重点行业、重点领域，把打击锋芒对准群众反映最强烈、最深恶痛绝的各类黑恶势力违法犯罪，依法打狠打准，打出声威，打出战果，打出群众的认可度、满意度。严打“两抢一盗”、电信诈骗、传销等发生在群众身边的违法犯罪，“两抢一盗”可防性案件发案同比下降20%。全面推进立体化、信息化社会治安防控体系建设，继续实施“雪亮工程”，为133个老旧小区安装视频监控，10月底前完成7500个视频监控点建设，实现市主城区视频监控全覆盖。推进“网格化”巡控体系建设，主城区增设警务站亭160个，提高群众的见警率、安全感。不断规范市县乡村四级退役军人管理服务机构，不折不扣落实军队退役人员政策，切实建成退役军人之家。妥善处理拖欠农民工工资问题。全力做好全国“两会”等重大活动安保维稳工作。

防范化解重大风险。建立健全地方金融监管工作机制，强化属地监管责任，细化行业监管任务清单；搭建地方金融监管和风险监测预警系统，强化宣传引导和奖励举报，提高风险源头管控能力；深入组织开展金融风险排查和清理整顿活动，依法稳妥处置金融风险点，严厉打击违法违规开办金融业务和非法集资、金融诈骗等违法犯罪行为，坚决守住不发生系统性、区域性金融风险底线。

同时，加强国防动员和国防后备力量建设。充分发挥工会、共青团、妇联等人民团体桥梁纽带作用，做好新闻出版广电、民族宗教、外事侨务、人民防空、气象地震、防灾减灾、史志档案、援疆援藏等工作。

努力建设人民满意政府

答好时代课题，完成历史使命，必须自身过硬。我们一定以强烈的历史责任感、深沉的使命忧患感、顽强的意志品质，坚持刀尖向内、勇于自我革命，以政治态度回应时代问卷，以责任担当书写历史答卷，以优异成绩接受人民阅卷。

坚持以新思想引领方向。自觉以习近平新时代中国特色社会主义思想为指导，坚决把维护以习近平同志为核心的党中央权威和集中统一领导作为最高政治原则和根本政治规矩，把践行“四个意识”熔化在思想上、落实在行动上，把坚持和加强党的领导贯彻到政府工作全过程和各方面。扎实开展“不忘初心、牢记使命”主题教育，持续推进“两学一做”学习教育常态化制度化，把践行“三严三实”贯穿于全部工作生活。

坚持以新标准执政为民。走好新形势下的群众路线，把民心民愿作为第一信号，把安民利民作为第一选择，把民情民意作为第一标准。切实把人民群众所盼的，变成政府真干的，切实把政府所干的，变成人民群众真盼的。依靠群众的智慧办群众的事情，依靠群众的力量建人民政府。聚焦全面建成小康社会奋斗目标，着力解决发展中不平衡不充分问题，使人民获得感幸福感安全感更加充实、更有保障、更可持续。

坚持以新自觉依法行政。深入贯彻全面依法治国基本方略，加快推进新时代法治政府建设各项任务。加强重点领域政府立法，不断提高政府立法公众参与度。落实法律顾问制度和公职律师制度，建立决策咨询论证专家库，不断扩大全国规范性文件合法性审查试点创建成果，“三重一大”事项必须集体研究决定，进一步提升行政决策的公信力和执行力。深化行政执法体制改革，全面推进行政执法三项制度，促进严格规范公正文明执法。自觉接受人大法律监督、政协民主监督、社会舆论监督，认真办理人大代表建议和政协委员提案，重大事项向人大报告、向政协通报、向社会公布，广泛听取民主党派、工商联、无党派人士、人民团体意见，让人民监督权力，让权力在阳光下运行。

坚持以新作风提升效能。深入开展纠正“四风”和作风纪律专项整治，持之以恒克服形式主义、官僚主义，久久为功祛除享乐主义、奢靡之风。扎实开展重点工作督查，对表态多调门高、行动少落实差等问题问责追责。认真开展大学习、大调研，不断强化“八种本领”，切实做到“五个过硬”。深化简政放权，推进“互联网+政务服务”，打通政策落实中的“中梗阻”和“最后一公里”，最大程度利企便民。坚持“一分部署、九分落实”，撸起袖子加油干，苦干实干拼命干，以永不懈怠的精神状态和一往无前的奋斗姿态，坚决把中央和省委、省政府以及市委重大决策部署落到实处。

坚持以新定力廉洁从政。严格落实全面从严治党主体责任，加强政府系统党风廉政建设和反腐败工作。强力整治群众身边的“微腐败”问题，凡是群众反映强烈的问题都要认真对待、加以解决，凡是损害群众利益的行为都要坚决纠正、严厉追责。强化工程建设、资源开发、产权交易、扶贫脱贫、棚户区改造等重点领域和关键环节的监督执纪，坚决强化不敢腐的震慑、扎实不能腐的笼子、增强不想腐的自觉，努力建设一支信念坚定、为民服务、勤政务实、敢于担当、清正廉洁的公务员队伍。
